# Low‐calcium diet in mice leads to reduced gut colonization by *Enterococcus faecium*

**DOI:** 10.1002/mbo3.936

**Published:** 2019-09-30

**Authors:** Janetta Top, Antoni P. A. Hendrickx, Marleen T. J. van Ampting, Kees van Limpt, Jan Knol, Denise van de Kamer, Johanna C. Braat, Marco Viveen, Malbert R. Rogers, Hans Kemperman, Rob J. L. Willems, Fernanda L. Paganelli

**Affiliations:** ^1^ Department of Medical Microbiology University Medical Center Utrecht Utrecht The Netherlands; ^2^ Danone Nutricia Research Utrecht The Netherlands; ^3^ Clinical Chemistry and Hematology University Medical Center Utrecht Utrecht The Netherlands

**Keywords:** *Enterococcus faecium*, gut colonization, low‐calcium diet, mouse model

## Abstract

The aim of this study was to determine whether dietary intervention influenced luminal Ca^2+^ levels and *Enterococcus faecium* gut colonization in mice. For this purpose, mice fed semi‐synthetic food AIN93 were compared to mice fed AIN93‐low calcium (LC). Administration of AIN93‐LC resulted in lower luminal Ca^2+^ levels independent of the presence of *E. faecium*. Furthermore, *E. faecium* gut colonization was reduced in mice fed AIN93‐LC based on culture, and which was in concordance with a reduction of Enterococcaceae in microbiota analysis. In conclusion, diet intervention might be a strategy for controlling gut colonization of *E. faecium,* an important opportunistic nosocomial pathogen.

## Graphical abstract

This study describes that dietary intervention based on low‐calcium food in mice results in lower luminal calcium concentrations, lower relative abundance of specific Gram‐positive families was observed, and reduction of *Enterococcus faecium* gut colonization in mice.

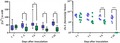

## 1 BACKGROUND

Over the last 30 years, *Enterococcus faecium* has emerged as an important multi‐resistant pathogen and the leading cause of nosocomial infections (Guzman Prieto et al., 2016). As gut colonization mostly precedes infections, it has been observed that *E. faecium* can become the dominant species in the gut of immune‐compromised patients who are treated with antibiotics (Ruiz‐Garbajosa et al., 2012). In a previous study, we used a mouse gut colonization model to show antibiotic‐mediated intestinal proliferation of *E. faecium* and the effects on the intestinal architecture, including an altered mucus‐associated gut bacterial layer and reduced colon wall and mucus thickness (Hendrickx et al., 2015). In addition, an increase of Ca^2+^ concentration in fecal water was observed, possibly as a reflection of an altered intestinal pathophysiology (Hendrickx et al., 2015). Furthermore, using monolayers of human colon HT‐29 cells, increasing concentrations of Ca^2+^, but not Mg^2+^ or K^+^, resulted in the cleavage of epithelial cadherin (E‐cadherin) and deformation of adherence junctions. The observed increased Ca^2+^ concentration correlated with the presence of an extracellular matrix, in which *E. faecium* was entrapped after inoculation, that lead to a higher recovery of *E. faecium* from the HT29 cells, while a similar extracellular matrix was observed in ceca and colons of mice treated with antibiotics and inoculated with *E. faecium* (Hendrickx et al., 2015). These observations suggest that elevated intraluminal Ca^2+^ concentration may act as a signaling molecule in the gut during antibiotic treatment and contribute to the formation of bacterium‐host molecule agglutinates that promote *E. faecium* colonization.

The aim of the current study was to investigate whether a low‐calcium diet reduces intestinal Ca^2+^ concentration and whether this also results in reduced gut colonization of *E. faecium* in mice.

## 2 METHODS

### 2.1 Bacterial strain

*Enterococcus faecium* strain E1162 was grown as described previously (Hendrickx et al., 2015).

### 2.2 Mouse intestinal colonization model

**Figure A1:**
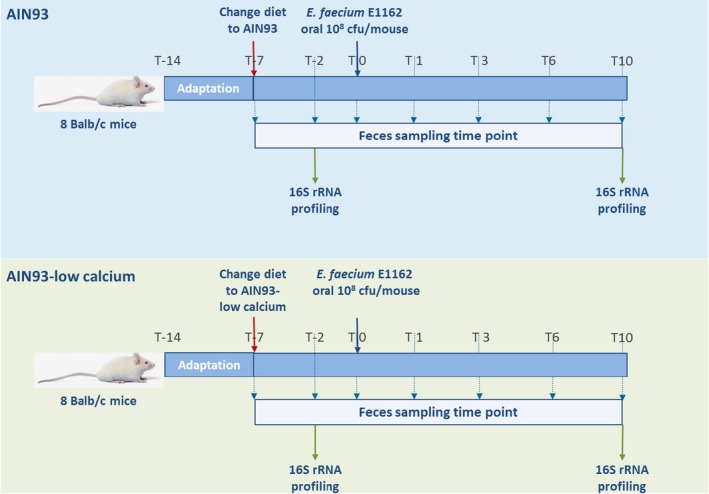
After 1 week of adaptation, two groups of 8 BALB/c mice changed to AIN93 and AIN93‐low calcium diet, respectively (day ‐7). At day 0, mice were orally inoculated with 10^8^ CFU *Enterococcus faecium* E1162 and sacrificed at day 10. Fecal pellets were collected at different time points throughout the experiment to determine i) luminal Ca^2+^ concentrations at day ‐7, ‐2, 0 (collected before inoculation with *E. faecium*), 1, and 10, ii) 16S rRNA profiling at day ‐2 and 10, iii) outgrowth of *E. faecium* at day 1, 3, 6, and 10. Mice were housed by 2, separated per specific diet (AIN93 or AIN93‐low calcium)

Sixteen specific pathogen‐free 10‐week‐old female wild‐type BALB/c mice (Charles River Laboratories) were colonized by *E. faecium* as previously described (Hendrickx et al., 2015) with the following modifications (Figure A1 in Appendix). Seven days prior to oral inoculation with *E. faecium*, the diet of the mice was switched from normal chow to semi‐synthetic food based on the purified diets for laboratory rodents as described by the American Institute of Nutrition (Reeves, Nielsen, & Fahey^1993^), i.e., AIN93 containing 5 g Ca/kg (8 mice) or a AIN93‐low calcium diet (AIN93‐LC, 8 mice) containing 0.1 g Ca/kg (ssniff‐Spezialdiäten GmbH). Within the same diet group, mice were housed 2 per cage. At day 0, all mice were orally inoculated with *E. faecium* (10^8^ colony‐forming units (CFU)/300 µl). Fecal pellets were collected at day ‐7, ‐2, 0 (collected before inoculation with *E. faecium*), 1, 3, 6, and 10 to (a) determine the number of *E. faecium* CFU on Slanetz‐Bartley agar plates (Tritium Microbiology B.V.) at days 1, 3, 6, and 10, (b) determine Ca^2+^concentrations in fecal extracts from these fecal pellets collected at day ‐7, ‐2, 0, 1, and 10 as described previously (Hendrickx et al., 2015), and (c) determine the microbiota composition by 16S rRNA gene sequencing (see below) on feces collected at day ‐2 and 10. The health status of the mice was regularly checked during the entire experiment, and no difference in health status was observed between the mice on the different diet regimes. None of the mice died during the experiment.

### 2.3 DNA isolation and 16S rRNA gene sequencing and analysis

Total DNA was isolated from the mice feces with the QIAamp Stool DNA mini kit (Qiagen) with small modifications. Two hundred milligrams of feces were transferred to 2 ml tubes containing 500 µl 0.1 mm Zirconium beads (Lab Services) and 900 µl ASL buffer (Qiagen). Bead beating was performed twice for 2 min at 3,500 beats/min with one interval of 2 min on ice using a BioSpec mini‐beadbeater‐24 (BioSpec products). Then the protocol was followed as recommended by the manufacturer's instruction. The 16S rRNA gene hypervariable regions V3 and V4 were amplified (469 bp) and sequenced with an Illumina MiSeq reagent Kit v3 (600‐cycle) on an Illumina MiSeq instrument (Illumina) (Fadrosh et al., 2014). Samples were analyzed with the QIIME™ 2 microbial community analysis pipeline (Caporaso et al., 2010). The generated sequence variant table and phylogenetic tree were established using QIIME's core_diversity_analyses.py workflow.

### 2.4 Statistical analysis

Cation measurements and colony‐forming unit (CFU) are expressed as mean ± *SEM* and median, respectively. The differences between groups or time points in these parameters were analyzed with a Mann–Whitney test using GraphPad Prism version 7.04.

The statistical framework analysis of composition of microbiomes (ANCOM) was used for the statistical analyses of the microbiota at family level (Mandal et al., 2015). R 3.5.0 in an environment of RStudio 1.1.383 (RStudio Team) was employed to calculate alpha diversity using the Shannon index, and significance was calculated by the Wilcoxon test. The global difference in microbiota composition, indicating beta diversity, was assessed using principal component analysis, employing zCompositions, centered log‐ratio (CLR) transformation, and ggplot R packages. PERMANOVA was performed using CLR transformed data. Homogeneity of data dispersion was checked using the betadisper package in R studio. When comparing the relative abundances of bacterial taxa, *p*‐values were adjusted for multiple comparisons using false discovery rates.

For all statistical analysis, we considered a *p*‐value lower than .05 as statistically significant.

## 3 RESULTS AND DISCUSSION

**Figure 1:**
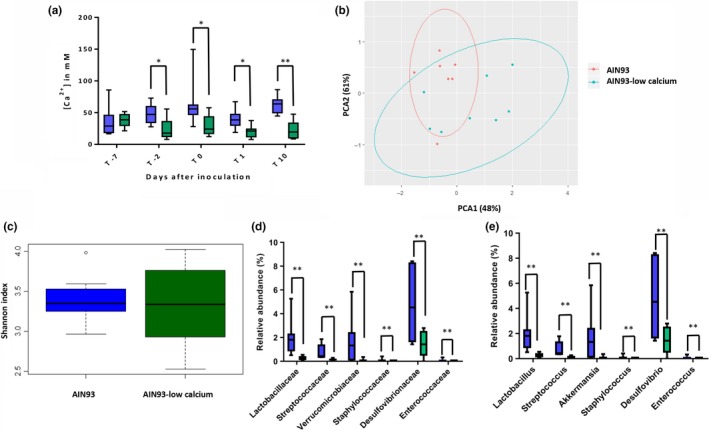
Diet interference effect on Ca^2+^ concentration in fecal water and microbiota composition 5 days after changing the diet regime (day ‐2). (a) Ca^2+^ concentration in fecal water. (b) Unsupervised PCA analysis at family level grouped (ellipse) and colored based on the two different diets regime (red: original AIN93 diet; blue: AIN93‐low calcium diet). (c) Shannon diversity representing total bacterial diversity in the 2 diet regiments. (d and e) Relative abundance of significant family (d) and genus (e) between AIN93 (blue) and AIN93‐low calcium (green) diets analyzed by Ancom. **p‐value < .05, **p‐value < .01

Luminal Ca^2+^ levels in fecal extracts from mice fed with AIN93 and AIN93‐LC diet were determined at different time points throughout the experiment (Figure 1a). After 7 days on the AIN93 or AIN93‐LC diet (day 0), a statistically significant (*p* = .026) lower luminal Ca^2+^ level was observed in mice on AIN93‐LC diet compared to mice on AIN93 diet; this difference became even more pronounced at day 10 (*p* = .001) (Figure 1a).

**Figure 2:**
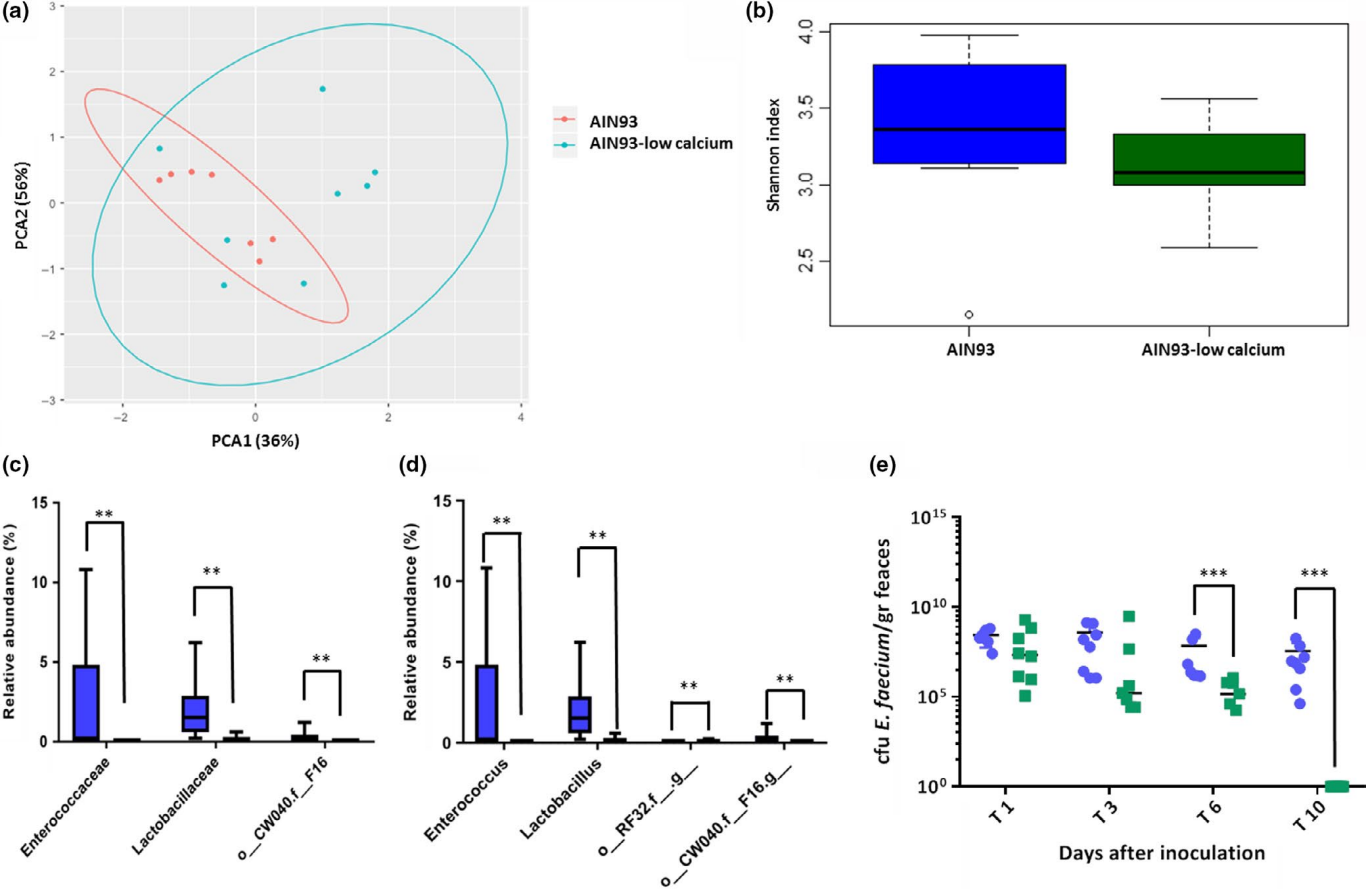
Microbiota composition after 10 days *Enterococcus faecium* colonization and determination of *E. faecium* colony‐forming unit (CFU) in feces at different time points in mice on AIN93 (in blue for b, c, d, and e panel) and AIN93‐low calcium (in green for b, c, d, and e panel) diet. (a) Unsupervised PCA analysis at family level grouped (ellipse) and colored based on the of 2 different diets regime (red: original AIN93 diet; blue: AIN93‐low calcium diet). (b) Shannon diversity representing total bacterial diversity in the 2 diet regiments. (c and d) Relative abundance of significant family (c) and genus (d) between the 2 diet regiments analyzed by Ancom. **p‐value < .01. D. *E. faecium* CFU counts in feces at different time points after *E. faecium* inoculation ***p‐value < .001

**Figure A2:**
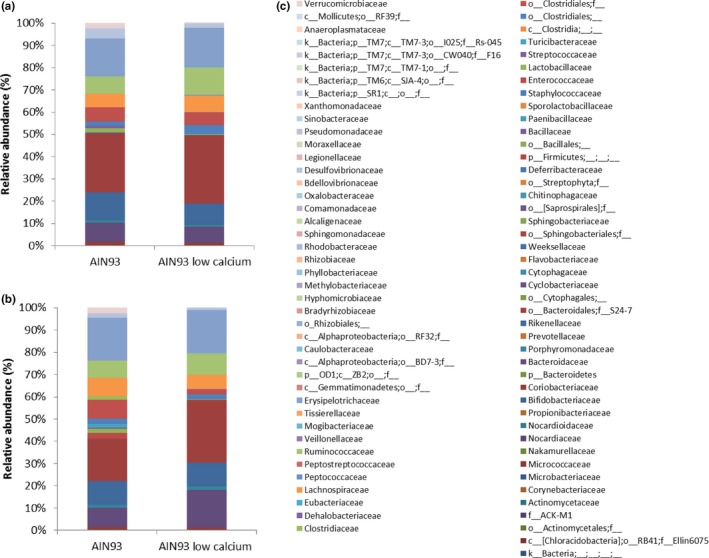
(a) Relative abundance of an average of 8 mice fed AIN93 and AIN93‐low calcium diet after 5 days of diet change (day ‐2). (b) relative abundance of an average of 8 mice fed AIN93 and AIN93‐low calcium diet 10 days after *Enterococcus faecium* inoculation (day 10). (c) Color indication for bacterial families

**Figure A3:**
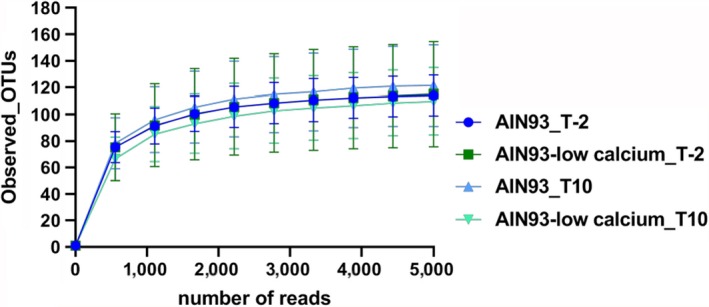
Average of observed OTUs with sequence depth of 5,000 reads of mice in the different diet regime. T‐2 represents mice 5 days after changing the diet. T10 represents 10 days after *Enterococcus faecium* inoculation

To determine the intestinal microbiota composition in fecal pellets from mice that were fed AIN93 and AIN93‐LC diet before and after inoculation with *E. faecium,* we performed 16S rRNA gene profiling on fecal samples obtained at day ‐2 and day 10 (Figure A1 in Appendix). Two mice were excluded from the analysis, because of the lower number of reads (237 reads) (AIN93 group T‐2) and insufficient feces for DNA isolation (AIN93‐LC 10 days after *E. faecium* inoculation). PCA analysis revealed differences in the microbiota 5 days after receiving the different diets, before *E. faecium* inoculation (day ‐2), with significant differences both before and after *E. faecium* inoculation, according to PERMANOVA test (*p* < .05) (Figure 1b and Figure 2a). At day ‐2, no difference was observed in the total (alpha) diversity and richness between the 2 groups (Figure 1c and Figure A3 in Appendix), while at day 10 the alpha diversity in mice on AIN93‐LC diet was lower (not significant) compared to mice on AIN93 (Figure 2b and Figure A3 in Appendix). A difference between the two time points was also observed in the relative abundance of bacterial families as determined by ANCOM. Enterococcaceae were only detected in the AIN93 group by 16S rRNA gene sequencing before *E. faecium* inoculation, however, in very low abundance (0.09%). No *E. faecium* was cultured from both groups before *E. faecium* inoculation, suggesting that the Enterococcaceae detected were not *E. faecium*. At day ‐2, the relative abundance of Lactobacillaceae, Streptococcaceae, Verrucomicrobiaceae, Staphylococcaceae, Desulfovibrionaceae, and Enterococcaceae (at genus level represented by *Lactobacillus*, *Streptococcus*, *Akkermansia*, *Staphylococcus*, *Desulfovibrio, and Enterococcus* (Figure 1e)) was significantly lower in mice fed AIN93‐LC relative to AIN93 (Figure 1d, 1e and Figure A2 in Appendix), while at day 10 mice on AIN93‐LC diet contained significant lower representatives from Lactobacillaceae, uncharacterized bacterial family o__CW040.f__F16 and Enterococcaceae compared to mice on AIN93 diet (Figure 2c and Figure 2b in Appendix). At genus level, *Enterococcus* and *Lactobacillus* were identified as significant, together with other two undetermined genera (o__CW040.f__F16 and order RF32) (Figure 2d). After *E. faecium* inoculation, the lower relative abundance of Enterococcaceae in mice on AIN93‐LC diet was in line with the *E. faecium* culture data (Figure 2e). Already at day 1 after *E. faecium* inoculation, CFU counts in the AIN93‐LC group were lower though not significant compared to mice fed AIN93. Lower CFU counts in the feces of mice fed AIN93‐LC were also observed at day 3 (not significant), though significant at day 6 (*p* = .0006). At day 10, we were not able to culture *E. faecium* from the feces of mice fed AIN93‐LC, while *E. faecium* colonization levels in mice fed AIN93 were still at the level of day 1 (*p* = .0002)(Figure 2d).

Also at day 10, a decrease in representatives of Verrucomicrobiaceae was observed in the gut microbiota of mice on AIN93‐LC diet compared to mice on AIN93 diet (Figure A2a,b in Appendix), although in contrast to day ‐2 this difference was not significant. Members of the family Verrucomicrobiaceae have been described as extremely sensitive for changes in chemical factors linked to soil fertility, including calcium (Navarrete et al., 2015). Apparently, this family present in the gut microbiome of mice is also sensitive to changes in calcium concentrations as observed by the decrease in the AIN93‐LC diet group. The observed decrease in Lactobacillaceae and Enterococcaceae families in the AIN93‐LC diet group seems to corroborate with previous findings where an increase of these families was observed in high calcium diets in pigs (Mann et al., 2014; Metzler‐Zebeli et al., 2013).

Caballero and coworkers identified a consortium of commensal bacteria, including the Clostridium cluster XIVa species, *Blautia producta,* and *Clostridium bolteae* that are implicated in colonization resistance against vancomycin resistance *E. faecium* (VRE) (Caballero et al., 2017). These bacterial species are part of the Lachnospiraceae family, which were indeed present in the microbiota of mice on AIN93 and AIN93‐LC diet, but no significant difference was observed in relative abundance between the two diet groups, suggesting that Ca^2+^ does not influence colonization resistance of the members of this specific bacterial family. Our hypothesize is that in our experiment the reduction in Ca^2+^ was the main factor to inhibiting *E. faecium* colonization. However, we cannot rule out that microbiota changes induced by Ca^2+^ may play a role in decreasing *E. faecium* colonization. To conclude, in the current study we demonstrate that luminal Ca^2+^ is reduced by a specific low‐calcium diet, that in mice fed a low‐calcium diet a lower relative abundance of specific Gram‐positive families was observed and that *E. faecium* intestinal colonization levels in these mice were reduced. This means that this work holds promise for future dietary interventions to reduce *E. faecium* gut colonization in hospitalized patients.

## CONFLICT OF INTEREST

None declared.

## AUTHOR CONTRIBUTIONS

Janetta Top, Antoni Hendrickx, Rob Willems, and Fernanda Paganelli conceived and designed the experiments and contributed to the writing of the manuscript. Marleen van Ampting, Kees van Limpt, and Jan Knol developed and provided the mouse food and contributed to the writing of the manuscript. Janetta Top, Denise van de Kamer, Johanna Braat, Marco Viveen, Malbert Rogers, and Hans Kemperman conducted the experiments.

## ETHICAL APPROVAL

This study was approved by the Animal Ethics Committee Utrecht and the Animal Welfare Body Utrecht as part of a project, which was licensed by the Central Authority for Scientific Procedures on Animals (CCD) (license number: AVD115002016568), which is the Dutch competent authority.

## Data Availability

The raw reads were deposited at the European Nucleotide Archive under the following project accession number PRJEB31853.
